# A Review of Current Research on the Use of Geopolymer Recycled Aggregate Concrete for Structural Members

**DOI:** 10.3390/ma15248911

**Published:** 2022-12-13

**Authors:** Muhammad Ahmed, Piero Colajanni, Salvatore Pagnotta

**Affiliations:** Department of Engineering, University of Palermo, 90128 Palermo, Italy

**Keywords:** geopolymer cement, recycled aggregate, structural behavior, compressive strength, flexural strength

## Abstract

Geopolymer cement (GPC) is a sustainable alternative to ordinary Portland cement (OPC) that considerably cuts the emission of carbon dioxide linked to the building of concrete structures. Over the last few decades, while a large number of papers have been written concerning the use of GPC with natural aggregates and OPC with recycled aggregates, few papers have been devoted to investigating the use of Geopolymer Recycled Aggregate Concrete (GRAC) in structural members. Most of them show more interest in the mechanical strength of the material, rather than the structural behavior of RC members. This review critically compiles the present and past research on the behavior of structural members cast with different types and compositions of GRAC. The focus is on the few research studies investigating the structural behavior of GRAC elements, with an analysis of the load-bearing capacity, the load-deflection mechanism, shear behavior, tensile and flexural strength, and ductility of GRAC structural members. This review aims to indicate the research and experimental tests needed in the future for characterizing the behavior of structural members made up of GRAC.

## 1. Introduction

Ordinary Portland Cement (OPC) concrete is the world’s most common and most widely used binding constructional material. It has a number of advantages as it is easily available, has a low cost, and is durable. However, this cement is criticized due to the emission of carbon dioxide during its manufacturing process. The production of OPC accounts for 5% of the world’s CO_2_ emissions; one ton of ordinary Portland cement releases approximately 0.9 tons of CO_2_ [1,2].

Moreover, the production of normal concrete requires a lot of natural aggregates, and there are environmental impacts as a result of aggregate extraction, including conversion of land use, erosion, loss of habitat of different species, etc. [3]. At the same time, a massive amount of construction waste is produced every year due to the demolition of buildings and other concrete structures [4].

To mitigate the environmental pollution due to the construction industry and the excessive use of natural resources to produce OPC, the need arises for an alternative to OPC made using natural waste materials, especially industrial by-products (i.e., slag). In addition, it must also be efficient in terms of cost and characteristics [5].

The scientific community and the development sector are credited with developing Geopolymer Cement (GPC) and the latest research shows that it is now one of the most reliable alternatives to conventional constructional binding materials, i.e., OPC [6,7].

The material used for manufacturing GPC is mainly of geological origin and has semicrystalline or amorphous aluminosilicate polymeric network structures. However, different compositions and types of materials can be used for GPC production [8]. GPC production involves mixing an optimum quantity of source materials and alkali activators, then curing the prepared mixture at low or high temperatures. The source materials can be metakaolin or coal-fired fly ash [9].

GPC can also be produced when waste material such as fly ash, Ground Granulated Blast Furnace Slag (GGBS), and clay containing aluminosilicate minerals are treated with an alkali solution such as sodium hydroxide. The alkali solution helps reduce the setting time of geopolymer cement [10].

GPC is promising as an effective alternative to OPC as it could limit the emission of CO_2_ gases and help reduce construction demolition waste [11]. Apart from being eco-friendly, the use of GPC instead of OPC results in lower production costs with comparable mechanical properties [12].

Geopolymer cement with aggregates produces geopolymer concrete. Due to the compelling properties of geopolymer concrete, it is now used for various applications in the construction sector, i.e., multifunctional plastering, and for thermal insulation [13]. It can also be used for soil stabilization or coastal infrastructures [14].

The concept of a circular economy, which is playing an increasingly prominent role in the definition of sustainable construction techniques and materials, promotes the use of Recycled Aggregates (RA) for concrete production. The use of RA obtained from Construction and Demolition Wastes (CDW) in the concrete industry can help preserve natural aggregate resources and will reduce the need for landfill space, making the construction industry more environmentally friendly and sustainable. The use of recycled aggregates taken from CDW has more than two decades of tradition in the field of construction with OPC concrete [15], as has been proven by various researchers [16].

**Figure 1:**
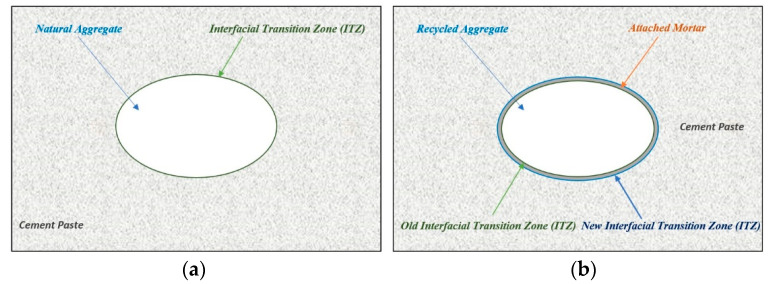
(**a**): Interfacial Transition Zone between NA and cement paste; and (**b**) Interfacial Transition Zone in the case of RA.

However, the properties of recycled aggregate are not as effective as those of natural aggregate. Indeed, the use of recycled aggregates for concrete structural members is hindered by the attached mortar since it has some negative effects on the strength of the mixture. It increases both the porosity of the recycled aggregate and the development of two different Interfacial Transition Zones (ITZ)s, between the recycled aggregate and new mortar and between the new mortar and attached mortar (Figure 1). The ITZ between new and old mortar is the weak zone that causes the reduction in strength [17].

That is the reason behind the greater uncertainty about the mechanical properties of concrete with RA, compared to that with NA, which limits the use of RA in OPC for non-structural applications or structural use with a low replacement ratio. However, RA efficiency can be significantly improved by treating the recycled aggregate with chemicals, heat, and abrasion [18].

Geopolymer concrete makes it possible to use a larger amount of RA, as demonstrated by research conducted in [19], where, with 100% use of recycled aggregates, concrete compressive strength values in excess of 45 MPa were achieved, and thus it is certainly compatible with the structural use of Geopolymer Recycled Aggregate Concrete (GRAC).

Le and Bui (2020) determined that the old concrete obtained from demolished construction waste can be mechanically crushed, sieved, cleaned, and sometimes also chemically treated in order to obtain RA for structural GRAC [17]. These recycled aggregates can be used in concrete as a partial substitute or full substitute depending upon the requirement [20].

In this regard, it has to be pointed out that there is no unanimous consensus on the influence of the compressive strength of the concrete used for the extraction of recycled aggregates on the strength of the GRAC to be obtained, as some authors state that the latter depends on the strength of the original concrete [21], and others only on the quality of the aggregates [22,23].

To study the environmental benefits of GPC, Almutairi et al. (2021) conducted comprehensive research and found that the use of geopolymer cement in the construction industry will reduce 80% of carbon dioxide emissions associated with the production of concrete. It will also be helpful in reducing the cost of raw materials required to produce concrete [24].

There is already significant ongoing research on the use of OPC with RA in structural members, and some on GPC with NA, but there is very little research on the behavior of structural members made of GRAC. The available data also provide conflicting results and assessments on the efficiency of GRAC. To begin, this review paper briefly describes the past and current research developments on the characterization of the mechanical properties of GRAC and then focuses on the few experimental research papers investigating the behavior of GRAC structural members, rather than the material itself.

## 2. Research Development on the Mechanical Properties of GRAC

Although research on making concrete from GPC has been going on for almost a decade, no guidelines are yet available for making geopolymer concrete, and currently, no empirical model is available for the reliable prediction of the compressive and tensile strength of GRAC, since they depend on several factors such as binder types, aggregate types, the molarity of the alkaline solutions, mixing procedure, casting temperature, and environmental conditions [25].

This section analyzes some of the research conducted by various authors on the mechanical strength of GRAC, focusing more on the use of different materials and the RA replacement ratio for improving the main mechanical characteristics of GRAC that influence the behavior of structural elements, such as the compressive strength, workability, and tensile and flexural strength. The studies are presented according to the type of material used for the preparation of GPC.

### 2.1. Fly Ash (FA)

Early GPC production techniques were based upon the use of FA, which is an efficient binder, due to its richness in silica and alumina, and provides cement with high compressive strength, especially when it is cured at a high temperature.

**Figure 2:**
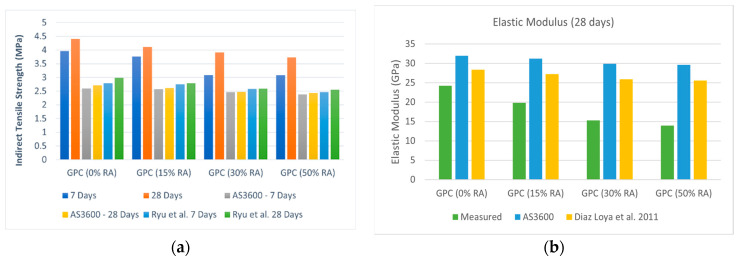
(**a**): Indirect tensile strength of GPC containing different percentages of NA and RA [27]; and (**b**) elastic modulus of GPC containing NA and RA [25,28].

Uddin Ahmed Shaikh (2016) did an experimental study to discover the durability and mechanical properties of GRAC made with FA (17% By Weight (BW)), sodium silicate (5% BW), and sodium hydroxide 8 M (2% BW). RA was used as a partial replacement (15%, 30%, and 50%) of NA. GPC with 100% NA was used as a reference for comparison. The test results showed that with an increase in RA content, the compressive strength, indirect tensile strength, and elastic modulus of geopolymer concrete decreased whether the test was performed after 7 or 28 days (Figure 2a) [25].

Moreover, it was found that the existing empirical models for OPC ((AS3600) [26]) and for GPC (Ryu et al. [27], Diaz Loya et al. [28]) containing natural aggregates underestimate the indirect tensile strength and overestimate the elastic modulus of GRAC (Figure 2b).

Nuaklong et al. (2016) performed a similar experimental study to find the effect of NaOH concentration (8 M, 12 M, and 16 M) on GPC prepared with FA and sodium silicate. Six different cylindrical samples with a 100 mm diameter and 200 mm height were prepared. Three of the samples had 100% limestone (as the natural aggregate) and three samples had 100% RA. The mechanical properties of GRAC prepared with FA (19% By Weight (BW)), sodium silicate (6.9% BW), and sodium hydroxide 8 M, 12 M, and 16 M (4.5% BW) were investigated.

The results showed that RA can be used in GPC with high calcium fly ash content. In the case of 8 M NaOH, the compressive strength of GRAC was found to be approximately 76.93% of GPC with NA, and 93% and 91% for 12 M NaOH and 16 M NaOH, respectively. In the case of 8 M NaOH, the flexural strength of GRAC was found to be approximately 95% of GPC with NA, while an increment of 4% and 7% was observed for 12 M NaOH and 16 M NaOH, respectively. Finally, 12 M NaOH was found to be most appropriate for GPC with high calcium fly ash content [29].

Wongsa et al. (2020) conducted comparative research to discover the physical properties of Pressed Geopolymer Concrete (PGC) made with GPC. The GPC was prepared with lignite coal fly ash, a sodium hydroxide solution (NaOH), and a sodium silicate solution. The GPC was mixed with RA obtained from demolition waste, Recycled Concrete Block Aggregate (RB), and limestone dust to obtain concrete to be used for concrete blocks. The RA and RB meshed into fine aggregates having a 4.75 mm diameter.

The results showed that: (a) pressed geopolymer concrete made up of limestone dust exhibited more compressive strength, less porosity, and water absorption than concrete made up of RB and RA; (b) the compressive strength of pressed GPC made up of RA and RB was nearly equal to the strength of moderate strength lightweight concrete prepared according to ACI 213 [30]; and (c) it was recommended that the pressed GPC with RA can be used not only for structural applications but also to make hollow geopolymer-based concrete blocks with better thermal insulation than cement-based concrete blocks [31].

Le and Bui (2021) studied the use of GRAC and the effect of the ratio between alkaline activated solution (AAS) and FA and the use of the lignosulfonate superplasticizer. AAS was taken as a combination of NaOH and a sodium silicate solution. Three different AAS/FA values (0.4, 0.45, and 0.5) were used for testing. An alkali-activated binder (geopolymer binder) was made using low calcium FA, a sodium silicate solution, a sodium hydroxide solution, and a lignosulfonate superplasticizer. Specimens were cured both at 60 °C and environmental temperature.

The results showed encouraging behavior of the GRAC specimen when the use of low calcium fly ash was joined with curing at 60 °C, even if a 100% replacement NA with RA was made, as a much lower decrease in strength was observed [32].

### 2.2. FA+GGBS (Flyash Combined with GGBS)

The use of GGBS, in addition to FA, increases the amount of aluminosilicate content, increasing the compressive strength and modulus of elasticity, and making them less dependent on the Water to Binder ratio (W/B). Moreover, it decreases the negative effect of weak ITZ that affects RA strength, resulting in a higher compressive strength.

Hu et al. (2019) performed an experimental study to find the flexural strength of GRAC members using different percentages of Ground Granulated Blast Furnace Slag (GGBS) and recycled aggregates. Twelve different mixtures were prepared. NA replacement was prepared at ratios of 0%, 50%, and 100% by weight. Specimens of size 100 × 100 × 400 mm^3^ were prepared and tested under a three-point loading test.

It was found that the use of GGBS provides an increment of compressive strength irrespective of the replacement ratio of RA, up to 100% when 30% of GGBS was used. This was due to the increase in high calcium content after GGBFS addition, which ultimately resulted in the formation of a gel phase that reduced the porosity.

The flexural strengths of the mixtures with recycled aggregate were found to be lower than that of the corresponding mixtures with natural aggregate. A further decrease in strength was observed with an increase in the RA percentage. The reason for the lower flexural strength was the poor quality of the recycled aggregate and the low bonding strength between the RA and the GPC. However, the flexural strength tended to increase with the addition of GGBS. After the inclusion of 10%, 20%, and 30% GGBS, the flexural strengths increased by 54%, 78%, and 92%, respectively, for the mixtures with 100% RA, and 51%, 60%, and 64%, respectively, for the mixtures with 50% RA [33], stressing that the addition of GGBS is more efficient when concrete with a 100% RA replacement ratio is considered.

Xie et al. (2019) conducted an experimental investigation to find the combined effects of FA and GGBS and considered the effect of W/B on the fresh and hardened properties of GRAC. A total of 100% RA was used for the preparation of GRAC, with different percentages of GGBS, FA, and the W/B ratio. The results revealed that the combination of GGBS and FA provides encouraging results regarding workability and the mechanical performance of GRAC.

Replacing the OPC matrix with FA/GGBS-based geopolymer improved the strength of recycled aggregate concretes. The compressive strength of GRAC was found to increase with a decrease in the W/B ratio, and with the inclusion of more GGBS content, the compressive strength increased. The compressive strength of GRAC with 50% and 75% GGBS content was found to be 50% and 180% higher than that of normal concrete [34].

Srinivas and Abhignya (2020) observed that by using FA and GGBS as a replacement for cement and RA as a replacement for NA, GRAC beams, and columns, performed much better than conventional reinforced concrete beams and columns referring to compressive and flexural strength. The optimum replacement percentage of RA was found to be 30% because with this replacement the ductile nature of both geopolymer and conventional concrete beams was almost the same [35].

Moulya and Chandrashekhar (2022) performed an experimental study to find the effect of the recycled aggregate replacement ratio on the strength of GRAC. The geopolymer concrete was made with a fixed ratio of GGBS to FA (50:50), and a sodium hydroxide concentration of 8 M was used. NA was replaced with RA at percentages of 0%, 10%, 20%, 30%, 40%, 50%, 60%, 70%, 80%, 90%, and 100%, and tests were performed after 3, 7, 14, and 28 days.

The results indicated that as the percentage substitution of RA increased, the compressive strength of the GRAC was reduced, but it was also found that the strength of GRAC increased with age/casting days. The maximum compressive strength of GRAC was found to be 60.02 MPa after 28 days with 70% RA replacement, which was very close to 100% natural aggregate geopolymer concrete. Hence, in this experimental analysis, it was recommended that a combination of FA and GGBS be used, with 8 M NaOH and 70% RA replacement for precast construction with environmental curing [36].

### 2.3. Metakaolin (MK)

Recently, the use of MK as a binder in GPC has been growing, since it is characterized by a high alumina and silica content and high reactivity due to its pozzolanic nature. Its high reaction speed with calcium hydroxide produces calcium aluminates hydrates and silicon aluminates that reduce the percentage of voids in concrete, improving its mechanical behavior.

In (2018) Nuaklong et al. (2018) modified the former mixture for GPC using MK with fly ash-based geopolymer concrete and performed a comparative assessment of the use of NA and 100% RA concrete. Two different schemes were adopted using: (1) Limestone as the natural aggregate in geopolymer concrete; (2) 100% recycled aggregate in geopolymer concrete. It was found that when the metakaolin amount was increased, the compressive strengths of GRAC with metakaolin (0, 10, 20, and 30%) were 32.9, 40.4, 45.0, and 47.2 MPa, respectively. GRAC mixtures with metakaolin achieved approximately 15–34% higher compressive strength than concrete without metakaolin. This enhancement of compressive strength was due to increased geo-polymerization and denseness of the microstructure. Increasing metakaolin from 10% to 30% also led to an increase in the splitting tensile strength from 2.9 to 5.4 MPa for GPC with NA and from 2.7 to 3.5 MPa for GRAC. The strength of geopolymer concrete also increased in both types (1 and 2) since the compressive strength of (1) was almost 7–19% higher than (2). Moreover, the researchers stressed that usually, the formation of geopolymer occurs by casting geopolymer slurry in the mold with a significant amount of alkali solution, increasing the chances of high porosity. These pores can act as a point of stress concentration and mechanical failure. In this context, the application of pressure reduces porosity [37].

Muduli and Mukharjee (2019) conducted an experimental study on the flexural strength of members made of GRAC. A total of 15 samples of size 100 × 100 × 500 mm^3^ were prepared with different percentages of MK and RA. Flexural strength tests were performed 28 days after casting. It was found that the sample without RA and MK had a flexural strength of 4.59 MPa, which decreased to 4.22 MPa and 3.9 MPa with the addition of 50% and 100% RA, respectively, in the concrete mix. The reason for the reduction was found to be poor bonding between RA and GPC and the presence of loose residual mortar attached to RA. MK proved to be efficient for the increase in flexural strength. For members having 50% RA, flexural strength gains of 2.8%, 8.1%, 9%, and 4.7% were observed with the incorporation of 5%, 10%, 15%, and 20% metakaolin, while 4.6%, 11.3%, 13.8% and 10% flexural strength gains were detected for concrete with 100% RA [38]. These results prove that MK is more efficient in increasing tensile strength, even with the higher replacement ratio of recycled aggregates.

Lee et al. (2020) stressed that superplasticizers can be used to improve the workability of concrete, but when they are applied to calcium-rich, alkali-activated materials, they give inconsistent results; by contrast, the use of Methyl Isobutyl Carbinol (MIBC) and polycarboxylate superplasticizers is found to be effective in improving the workability and strength of MK-based geopolymers, especially after 7 days [39].

Berhanul et al. (2021) examined the effect of metakaolin as a cement replacement on the properties of fresh and hardened recycled aggregate concrete and natural aggregate concrete. The recycled aggregates were obtained from first-hand cast laboratory cubes whose compressive strength was already known.

Different concrete mixtures were prepared and tested with different percentages of recycled aggregates and MK. Namely, 0%, 6%, 12%, 18%, and 24% replacement of OPC with MK, and 0%, 25%, 50%, 75%, and 100% replacement of NA with RA was made. The results showed that the use of metakaolin as a cement replacement improved the strength of GRAC. In the case of GRAC with 100% replacement with RA, the optimum content for OPC replacement with MK was found to be 6% [40].

Xu et al. (2021) reviewed the current research on the mechanical properties of GRAC, confirming that GPC in GRAC is an ideal substitute for cement and, similarly, RA is also an ideal substitute for NA because of its environment-friendly effects. The strength of GRAC depends on many factors such as the type of geopolymer, the casting temperature, the type of aggregates, etc. But these strength-influencing factors are similar to those for GPC, and there is a lack of research on the use of GRAC so the focus must be on the practical use of GRAC [41].

### 2.4. Mixing Procedure

Treatments of RA to reduce the unfavorable influence on GRAC mechanical properties lie beyond the scope of this review. Here, we will just mention that to mitigate the risk of failure due to the ITZ of recycled aggregates, Liang et al. (2013) previously proposed two different mixing procedures, named the Mortar Mixing Approach (MMA) and the Sand-Enveloped Mixing Approach (SEMA).

**Figure 3:**
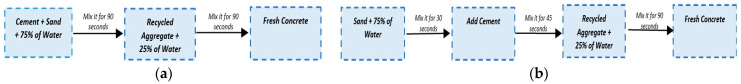
Schematic diagram of (**a**): the Mortar Mixing Approach (MMA) method; and (**b**) the Sand-Enveloped Mixing Approach (SEMA) method [42].

The schematic diagrams of both methods are given in (Figure 3). In the SEMA method, RA underwent pre-surface treatment 7 days before mixing, obtaining a higher 28-day compressive strength as compared to that of recycled aggregate concrete made with the MMA method. Due to these mixing processes, an additional layer of cement formed on the aggregate surface, which decreased its porosity as well as reduced its high-water absorption; this ultimately resulted in an improvement in strength. The results obtained showed that using MMA improved the compressive strength of concrete made with 100% coarse RA. However, these methods generate an increase in cost and casting time [42].

Recently, Alqarni et al. (2021) proposed a new two-stage mixing approach with silica fume and cement. In this treatment process, a cement-silica fume slurry solution was prepared by mixing the cement and silica fume in different percentages with water by weight. RA was dried in an oven for a day and then cooled. After that, the RA was mixed with a cement-silica fume slurry solution for about 30 min; this treatment was found to increase the compressive strength of concrete [43].

### 2.5. Comparison of Results

**Figure 4:**
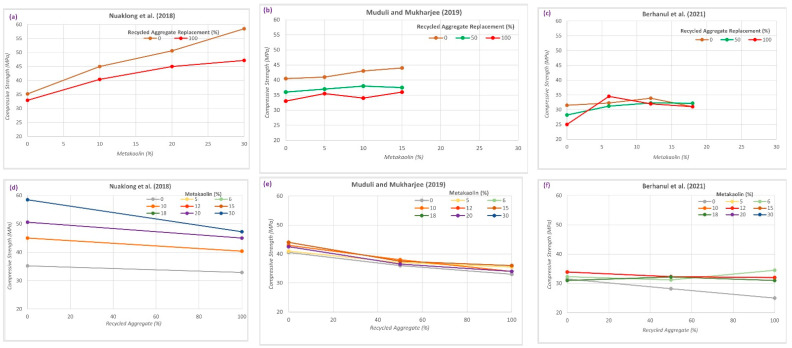
Relationship between the compressive strength of GRAC with different percentage contents of RA and MK. (**a**): compressive strength variation with different percentage of MK for 0% and 100% RA replacement [37]; (**b**): compressive strength variation with different percentage of MK for 0%, 50% and 100% RA replacement [38]; (**c**): compressive strength variation with different percentage of MK for 0%, 50% and 100% RA replacement [40]; (**d**): compressive strength relation with different percentage of RA for different percentage of MK [37]; (**e**): compressive strength relation with different percentage of RA for different percentage of MK [38]; (**f**): compressive strength relation with different percentage of RA for different percentage of MK [40].

Figure 4 shows a comparison of the results reported by three different researchers, namely, Nuaklong et al., 2018 [37], Muduli and Mukharjee (2019) [38], and Berhanul et al., 2021 [40], showing the effect of metakaolin increases and/or the percentage of recycled aggregate replacement on the compressive strength of GRAC.

Figure 4a,b shows that the larger the replacement with MK, the larger the increase in the compressive strength; surprisingly, the increase is more evident for NA than for RA. By contrast, the results of Berhanul et al. (2021) [40], shown in Figure 4c, show an optimum MK replacement ratio, and the larger the recycled aggregate replacement ratio, the smaller the value of the optimum MK replacement ratio.

This trend is not reflected in most of the other research in the literature. Figure 4d,e shows the generalized reduction in the compressive strength with an increase in the RA replacement ratio; interestingly, the larger the MK content, the smaller the compressive strength reduction due to the use of RA.

**Figure 5:**
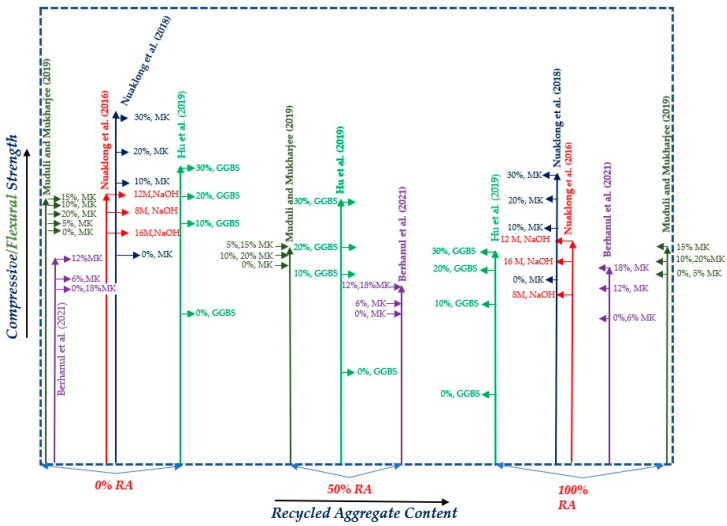
Comparative overview of previous research on GRAC strength with the inclusion of different percentages of RA and other materials (MK and GGBS) [29,33,37,38,40].

**Figure 6:**
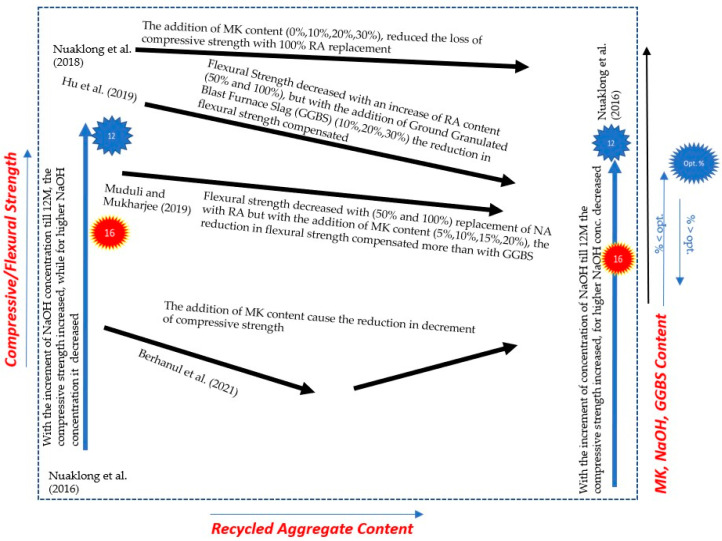
General overview of previous research on GRAC [29,33,37,38,40].

A more comprehensive comparison of the different approaches to compensating the strength reduction due to the incorporation of significant amounts of recycled aggregates is reported in Figure 5, where the results of tests on both compressive and flexural strength (green line) are reported for different binders and recycling aggregate replacement ratios. From the discussion above and by critically analyzing the data represented in Figure 5, it can be seen that in the past, the strength and properties of GPC were not enough to incorporate a large percentage of recycled aggregates. Hence, low-quality GRAC was produced with less strength. However, with the modification in the chemical composition of GPC, i.e., by the addition of MK mixed with a NaOH solution with different concentrations and GGBS, a significant improvement was observed in the strength of GRAC, and encouraging results were obtained. In most studies, it was found that the strength was increased with the addition of more MK and GGBS, while it was reduced with the addition of more RA. However, in Nuakalong et al. (2016) [29], an optimal value of NaOH was found to be 12% while in Berhanul et al. (2021) [40], with MK, it was found to be 6%. The above results are qualitatively represented in Figure 6.

## 3. Research Progress on the Structural Members Made up of GRAC

This section focuses on the structural behavior of GRAC members, reviewing recent research developments on flexural strength, the load-bearing capacity, the load-displacement relationship, and the shear behavior of GRAC structural members. The results are discussed on the basis of the results reviewed in the previous section. Most of the research is devoted to investigating the flexural behavior of GRAC-reinforced beams with fixed concrete properties and the RA replacement ratio being varied, while only one paper investigated the behavior of a shear critical beam [44], and another investigated the behavior of a reinforced column under an axial load. Lastly, one paper (Romanazzi et al. (2022) [45]) is devoted to investigating the bond-slip relationship of reinforced GRAC elements.

Thangamanibindhu and Murthy (2015) carried out research on the behavior of environment-cured GRAC beams. The GPC was prepared using GGBS, FA, and sodium silicate solution. Sodium hydroxide was used as an alkali activator, and a superplasticizer was used to improve the mechanical characteristics. A total of nine beams were cast having a dimension of 100 × 200 × 1200 mm^3^, longitudinal and hanger reinforcement #2 with ⌀ = 10 mm, stirrups of ⌀ = 6 mm with a pitch of 100 mm, and tested in flexure. Three beams were prepared with conventional concrete mixes having a 0%, 10%, and 30% replacement of RA (1). Six beams had varying proportions of FA (12.6–8.33% BW), GGBS (4.2–8.5% BW), and recycled coarse aggregates (0%, 10%, and 30%) (2). A four-point loading scheme was used in the test. It was found that the average ultimate loads for GRAC beams ranged from 65 kN to 103.55 kN, while for (1) it ranged from 38.1 kN to 55.6 kN. Moreover, the cracking load of GRAC beams was found on average to be 30% more than that of conventional reinforced concrete beams. The load-carrying capacity of all the beams decreased when a larger quantity of recycled aggregates was incorporated. The same load-deflection characteristics were obtained for ordinary reinforced cement concrete beams and geopolymer concrete with 10% replacement of RA. The deflection for (2) ranged between 5.28 mm and 7.04 mm and for (1) it ranged between 3.35 mm and 4.54 mm. The failure behavior of geopolymer concrete beams was found to be similar to that of cement concrete beams, as both types of beams failed initially due to the yielding of the tensile steel; then concrete crushing occurred [46].

Kathirvel and Kaliyaperumal (2016) conducted an experimental study to investigate the influence of RA obtained from demolished construction waste on the flexural behavior of GRAC beams. The casting of GRAC occurred at room temperature. GGBS (19.71% BW), NaOH (3.28% BW), sodium silicate (6.5% BW), and superplasticizers were used to achieve high strength. A total of six beams with dimensions of 1.5 m × 0.1 m × 0.15 m, having #2 @ 12 mm ⌀ longitudinal bars, #2 @ 8 mm ⌀ hanger bars, and stirrups of 6 mm @100 mm c/c were cast. Five beams were of geopolymer concrete having 0%, 25%, 50%, 75%, and 100% replacement of RA, and one beam was normal concrete. All beams were tested under a four-point flexure load scheme similar to that indicated by ASTM C1161 [47]. RA was pre-wetted to mitigate the consequence of the rapid reduction in concrete workability.

**Figure 7:**
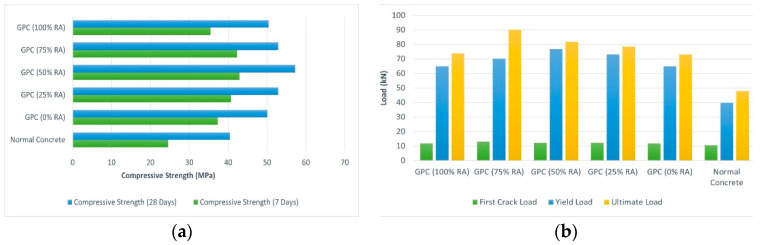
(**a**): The compressive strength of cylindrical specimens with different percentages of RA after 7 and 28 days; (**b**) the load-carrying capacity of beams at various RA replacement ratios [19].

The results revealed that with an increase in RA content, there was a slight decrease in initial stiffness. Due to pre-wetting and the inclusion of plasticizers up to 50% RA replacement, the compressive strength and water absorption characteristics improved (Figure 7a), while with the replacement of NA with RA up to 75% the load-bearing capacity of beams increased (Figure 7b). By contrast, it started to decrease after the replacement exceeded 75% [19].

Deepa and Jithin (2017) performed an experimental study to find the strength and behavior of GRAC beams. RA taken from demolition waste were used as coarse aggregate.

**Figure 8:**
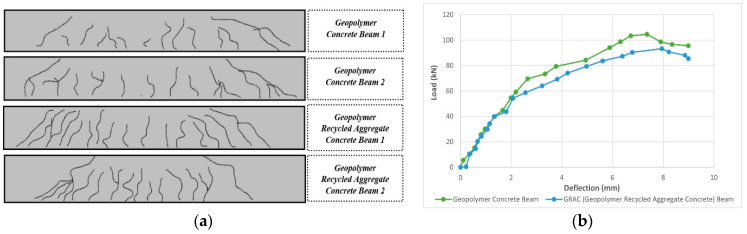
(**a**): The crack pattern of geopolymer concrete beams and GRAC beams; and (**b**) the load-deflection curves of geopolymer concrete beams and GRAC beams [48].

The ingredients of GPC were low-calcium FA (Class F), sodium silicate alkaline solutions, and a sodium hydroxide solution. Coarse aggregate, fine aggregate, and superplasticizer were used with GPC to prepare GRAC. NA were replaced with RA with the following percentages: 20%, 30%, 40%, 50%, and 60%. The optimum replacement ratio was found to be 40% on the basis of workability. Beams were cast of dimensions 175 mm × 150 mm × 1200 mm, having #2 @ 10 mm ⌀ and #1 @ 6 mm ⌀ longitudinal bars, #2 @ 6 mm ⌀ hanger bars, and stirrups of 6 mm @100 mm c/c. The beams were then subjected to a bending test. From the experimental study, it was concluded that there was a slight reduction in strength and deformability with the addition of RA (Figure 8b). The flexural strength of a GRAC beam with 20% RA replacement was 4% lower than that of a geopolymer concrete beam with 100% NA, while the reduction was 31% for the GRAC beam with 60% RA replacement. It was also observed that the GRAC beams showed a larger size and increased number of cracks as compared to normal geopolymer concrete beams (Figure 8a). This is due to the porous structure of the recycled aggregates which produces a reduction in the tensile strength of GRAC [48].

The main aim of the experimental study performed by Srinivas and Anhignya (2021) was to determine the optimum percentage of RA for GRAC and to study the behavior of structural members (beams and columns) when subjected to axial compression or bending. Recycled aggregates were obtained from demolition waste and mixed with geopolymer cement to produce GRAC. In the production of GRAC, GPC was prepared using fly ash (13.68% BW), GGBS (2.41% BW), and an alkaline solution (sodium silicate and sodium hydroxide). Naphthalene sulphonate formaldehyde and superplasticizers were used for better strength of GPC. In this experimental study, 20%, 30%, 40%, 50%, and 60% replacement of recycled aggregates was made. Three beams and three columns of dimensions 150 mm × 150 mm × 1200 mm were prepared. The beams were tested under a four-point loading test and the columns were tested under axial loading.

The results revealed that based on mechanical properties and workability, the optimum replacement percentage of RA was 40%. The crack pattern and failure mode of GRAC beams and geopolymer concrete beams were the same. GRAC columns with 40% of RA in axial compression behaved in the same way as geopolymer concrete beams. Due to the inclusion of naphthalene sulphonate formaldehyde, it was also found that almost 8% more ultimate load strength was obtained for GRAC as compared to geopolymer concrete beams; it was thus suggested that GRAC is a practical and eco-friendly solution [49].

Zhang et al. (2021) conducted a comparative study on GRAC and ordinary recycled aggregate concrete beams (OPC with RA). Static loading tests were conducted on three ordinary recycled aggregate concrete beams and seven GRAC beams. Metakaolin-based fly ash geopolymer and alkaline solution were used in the preparation of GPC. MK was used at 5.65% BW, while fly ash and potassium silicate were used at 5.65% BW. The test variables included the RA replacement ratio, the replacement pattern, and the reinforcement ratio. Three replacement ratios (30%, 70%, and 100%) of RA were taken. The conventional aggregate replacement pattern was to replace the same percentage of all particle sizes but in a new, larger replacement pattern; (up to 19 mm) NA particles were replaced with RA, and a 70% replacement ratio was used/set in both replacement patterns. Ten reinforced concrete beams with dimensions (1800 mm (L) × 100 mm (W) × 250 mm (H)) having the same geometry but different concrete types and replacement ratios were made. The bottom longitudinal reinforcement of eight beams was #2 @ 14 mm ⌀, one beam was #2 @ 10 mm ⌀, and one was #2 @ 18 mm ⌀. For all beams, the hanging bars were #2 @ 10 mm ⌀ and stirrups were 6 mm ⌀ @ 100 mm spacing.

**Figure 9:**
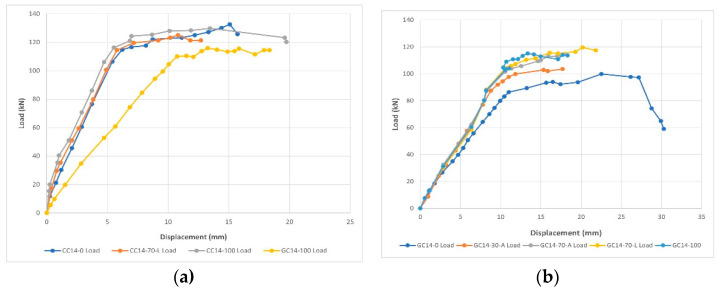
(**a**): The load-displacement curves of geopolymer and ordinary Portland cement beams; and (**b**): the load-displacement curves of GRAC with different ratios and replacement patterns [50].

The tests revealed that the geopolymer concrete has the same compressive strength as ordinary concrete but with a smaller elastic modulus (e.g., 28.9 GPa for ordinary and 10.2 GPa for geopolymer) because Young’s modulus of geopolymer concrete is affected by a microstructure based on speciation of the alkali silicate activating solutions as well as the properties of the aggregates. Because of this, GRAC beams have a lower height of the neutral axis and more deflection than ordinary recycled aggregate concrete beams at the same loading (Figure 9a), depending on replacement patterns. The ultimate deflection was found to be 17.9 mm for CC14-100 (ordinary concrete beam with longitudinal bars of 14 mm ø with 100% RA), 19 mm for GC14-100 (geopolymer concrete beam with longitudinal bars of 14 mm ø with 100% RA), 12.7 mm for CC14-70-L (ordinary concrete beam with longitudinal bars of 14 mm ø with a 70% replacement of large natural aggregates), and 21.9 mm for GC14-70-L (geopolymer concrete beam with longitudinal bars of 14 mm ø with a 70% replacement of large natural aggregates) (Figure 9b). GRAC beams also had a slightly lower cracking load, ductility, and bending capacity. It was also found that the cracking load and cracking moment of the GRAC with 100% RA were found to be approximately 23% lower compared to the ordinary concrete beam with 100% NA. In this study, a high alkali solution was used which reduced the elastic modulus. When the alkali concentration was reduced, the geopolymer concrete showed better results [50].

Aldemir et al. (2022) were the only researchers who investigated the shear behavior and structural performance of GRAC beams in detail. A new type of geopolymer concrete was prepared from demolition wastes. Roof tiles, red clay, hollow bricks, and concrete rubble were used as wastes along with slag, fly ash, sodium hydroxide, and sodium silicate. In this study, four different mixes were prepared: (1) GRAC, (2) geopolymer natural aggregate concrete, (3) ordinary recycled aggregate concrete, and (4) normal concrete. In previous studies, the authors found that with the addition of RA both in ordinary concrete and in GPC, workability and compressive strength decreased but, in this study, it was assessed that, when the same Water Cement Ratio (W/C) ratio is used, in GRAC the porosity of the concrete decreased because part of the water is absorbed by the RA, thus increasing the strength of the concrete. Three shear span-to-effective depth ratios (a/d = 0.5, 1, 1.65) were used to examine the different failure modes. Four beams of dimension 150 × 250 × 1100 mm^3^ were cast for each shear span-to-effective depth ratio and each concrete mix type. Then, 4-point bending tests were performed to determine the shear behavior of the beams. Parameters including load-deflection curves, moment curves, and crack propagation were used to assess the mechanical performance of the beams. It was also found that the compressive strength of the members made of this GRAC was 3% higher than that of conventional concrete members. The results indicated that the beams made up of geopolymer concrete exhibited a similar performance to normal concrete beams of the same grade. However, when recycled aggregates were used, then the failure mechanism shifted from flexure-dominated to shear-dominated. This shift was more common in the beams with a larger span to an effective depth ratio [44].

Raza et al. (2021) performed an experimental study on the structural performance of GRAC columns with glass fiber-reinforced composite bars and hooks subjected to a compressive axial load. Nine mid-scale circular columns of dimensions 250 mm × 1150 mm, having six, eight, or ten longitudinal reinforcing bars (reinforcement ratio of 1.57%, 2.11%, and 2.6%), and pitch hooks at 75 mm, 150 mm, and 250 mm (corresponding to a transversal reinforcement volumetric ratio of 1.42%, 0.71%, and 0.50%, respectively) were tested. The mix design of GPC was chosen with the following proportion by weight: RA 50.13%, sand 21.18%, water 5.20%, sodium hydroxide solution (14 M) 1.65%, FA 10.236%, GGBS 6.89%, superplasticizer Sika ViscoCrete-3425 0.16%, and sodium silicate 4.44%.

**Figure 10:**
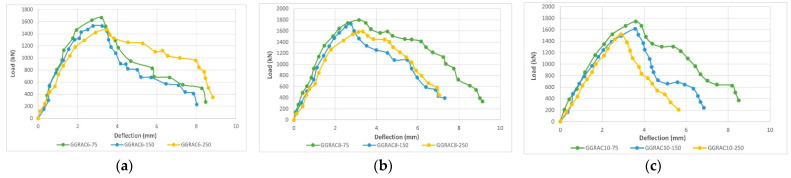
The load-displacement curves of GRAC columns with different reinforcement from glass fiber reinforced composite bars. (**a**): With 6 mm diameter bars and a hook pitch of 75 mm, 150 mm, and 250 mm, (**b**): with 8 mm diameter bars and a hook pitch of 75 mm, 150 mm, and 250 mm, and (**c**): with 10 mm diameter bars and a hook pitch of 75 mm, 150 mm, and 250 mm [51].

GRAC was prepared with the 100% replacement of NA. The axial force-displacement curves in Figure 10 stress the influence of the GFRP longitudinal reinforcement ratio and circular hoop spacing on the strength and deformation capacity of the GRAC. The authors noted that an increase in the number of longitudinal GFRP bars up to eight improved the axial load capacity of GRAC members, while a further increase to ten bars reduced the axial load capacity of the specimens. Reducing the hoop pitch from 250 mm to 150 mm produced an average increase in the axial load capacity by 6.3%, while a further reduction in the spacing to 50 mm produced a total gain of 1.13%. A noticeable increase in the ultimate deflection, i.e., the deflection of the post-peak softening branch at which the specimen attains 85% of its load capacity, was only found when ten vertical reinforcing bars were put in place (21% and 25% for pitch reduction from 250 mm up to 150 mm and 50 mm, respectively), while a clear trend was not revealed for six and eight longitudinal rebars [51]. The results prove that the effect of confinement provided by transversal reinforcement can be fully exploited to prevent the buckling of a longitudinal bar, i.e., the longitudinal bar diameter is large enough to avoid buckling phenomena with the chosen hook pitch.

The bond strength between the concrete and steel reinforcement is essential for the ultimate strength of the structural members. Romanazzi et al. (2022) performed an experimental investigation to examine the bond behavior of GPC with steel bars and sand-coated Glass Fiber Reinforced Polymer (GFRP) bars using a pull-out test. The test results revealed that the adhesion bond characterizing the behavior of GPC is stronger than that shown with OPC, both for sand-coated GFRP bars and deformed steel. However, the ultimate bond strength of GPC with steel bars was two to three times higher than that of sand-coated GFRP bars. This is due to the fact that there is adequate mechanical interlocking and a good bond strength between the GPC and steel bars, irrespective of the bar diameter. Thus, in the case of GFRP bars, the predominant mechanisms are those of adhesion and friction, while between concrete and steel bars, the predominant contribution is that of mechanical interlocking [45].

## 4. Discussion

- The use of geopolymer concrete with recycled aggregate is a complex topic. The efficiency of GRAC depends on several factors: the type and composition of GPC, the molarity of the alkaline solutions, the mixing procedure, the curing temperature and environmental conditions, and the mechanical and chemical characteristics of the RA and ITZ; how these factors relate to the amount and characteristics of the attached mortar; the extraction process from the demolition construction waste; and, lastly, the replacement percentage of RA with NA, etc.
- The type of geopolymer cement created is one of the most important aspects, as different amounts and types of chemicals are used in the production of GPC. In older studies, GPC was prepared with fly ash and the results were not very encouraging (the recycled aggregate replacement ratio was limited to 30–40%).
- In recent research, it was found that when metakaolin and GGBS-based GPC were used, larger values of replacement ratios (up to 100%) can occur without a significant reduction in the flexural strength of the structural member. The tensile strength of GPC concrete can increase the cracking strength of the beam, which decreases with an increase in the RA replacement ratio; however, OPC with NA-reinforced beams and GRAC-reinforced beams usually exhibit a similar failure mode and cracking pattern. Only a shear-critical beam can exhibit premature failure when GRAC with a large RA replacement ratio is used.
- A large amount of RA can cause workability issues, but from the latest research, it was also found that the use of polycarboxylate superplasticizers and methyl isobutyl carbinol (MIBC) can improve workability, allowing for a reduction in the W/B ratio and increasing the strength of metakaolin-based geopolymers.
- In this regard, it must be pointed out that most of the research was performed using RA in a saturated surface condition which reduces the compressive strength and elastic modulus of the concrete.
- The excessive use of alkali activators reduces the elastic modulus of concrete, causing an increase in beam deflection. Hence, a precise quantity of alkali activators in relation to other materials should be used in GPC production. There is not yet a unanimous consensus on the exact quantity and type of material; therefore, the quantity and type of GPC should be chosen depending on the characteristics of available RA.
- An Interfacial Transition Zone (ITZ) develops between the attached mortar and the new cement paste. This is one of the weakest zones, so proper chemical and mechanical treatment is advised before using RA. It was found in the latest research that the addition of fillers and fly ash is helpful to fill the pores of RA, thus reducing the vulnerability of failure along the ITZ; to this aim, the Two Stage Mixing Technique (TSMA) can also be adopted, in which a cement coating forms on the surface of the recycled aggregate, thus filling up the cracks before actual mixing of concrete.
- From the literature review, it is seen that there are no general limits to the use of coarse RA in a concrete mixture. Some of the former researchers recommended a maximum 30% replacement of NA with RA, while recent researchers suggest that the RA replacement can be up to 50% or 100% if the mix design, batching methodology, and moisture condition of the RA are properly handled. In most of the research, the use of a 70% RA replacement ratio does not significantly affect the load capacity and only slightly affects the deformability of the GRAC beam loaded in flexure.
- In general, much research is still needed to identify the optimal mix design and to optimize the production methods and rules for setting the geopolymer concrete and its mechanical properties, particle size distributions, and aggregate processing for the production of GRAC. However, current knowledge already makes it possible to produce GRAC with a predetermined class of compressive strength, while its tensile strength and related characteristics, such as the bond between GRAC and reinforcement, related cracking phenomena, and ductility that can be conferred through confinement, are more uncertain.
- Particular attention must be paid when GRAC is to be used in conjunction with GFRP bars, since the confinement of the transverse reinforcement, which is able to provide an increase in compressive strength, is not always effective in increasing the deformation at the decay of resistance to 85% of the maximum value, and, more generally, ductility and toughness, a condition which can only be achieved in the presence of an adequate number of longitudinal bars capable of transferring the confinement action exerted by the transverse reinforcement more uniformly.
- Regarding the bond between GRAC and reinforcement, GRAC has exhibited promising behavior when used in conjunction with steel cold-form reinforcement, while bond strength is reduced up to 33% when used with sand-coated GFRP bars. This is because the predominant influence ensuring the bond between concrete and steel bars is mechanical interlocking, while in the case of GFRP bars, the predominant mechanisms are those of adhesion and friction.
- It is also found that no research has been conducted on the beam-to-column joint made up of GRAC. The beam-column joint is one of the most vulnerable structural members belonging to moment-resisting frames made of cast-in-situ concrete. Thus, in order to prove the effectiveness of GRAC, this aspect should also be analyzed.
- Similarly, a research gap has been found regarding the seismic behavior of GRAC structural members. The seismic assessment of GRAC structural members should be carried out in order to evaluate the ductility and energy dissipation capacity of reinforced GRAC for construction in seismic areas.
- Therefore, from the above literature review, it can be concluded that there is still a need for experimental tests that study the behavior of structural members made up of GRAC, characterizing the phenomenon of bond, the strength and ductility of members subjected to bending with or without axial load, and the shear strength of members with and without transverse reinforcement. Moreover, there is a need for studies focusing on the influence of the casting and curing conditions on the mechanical strengths of the structural member.

## Data Availability

The data presented in this study are available on request from the corresponding author.

## Abbreviations

GRAC	Geopolymer Recycled Aggregate Concrete
OPC	Ordinary Portland Cement
GPC	Geo Polymer Cement
GGBS	Ground Granulated Blast Furnace Slag
RA	Recycled Aggregates
ITZs	Interfacial Transition Zones
NA	Natural Aggregate
MK	Metakaolin
HCFA	High Calcium Fly Ash
PGC	Pressed Geopolymer Concrete
RB	Recycled Concrete Block Aggregate
FA	Fly ash
MMA	Mortar Mixing Approach
SEMA	Sand-Enveloped Mixing Approach
GFRP	Glass Fiber Reinforced Polymer
TSMA	Two-Stage Mixing Technique
BW	By Weight
W/B	Water to Binder ratio
MIBC	Methyl Isobutyl Carbinol
W/C	Water Cement Ratio
